# DNA damage in canine leishmaniasis infection is detectable by micronucleus and comet assay in peripheral blood samples

**DOI:** 10.29374/2527-2179.bjvm001425

**Published:** 2025-06-13

**Authors:** Roberta Tognareli Ruiz, Aline Cechinel Assing Batista, Jorge Luis Maria Ruiz

**Affiliations:** 1 Laboratório de Biotecnologia Aplica à Saúde. Universidade Federal da Integração Latino-Americana – UNILA, Foz do Iguaçú, PR, Brazil; 2 Programa de Pós-graduação em Patologia Experimental e Comparada. Universidade de São Paulo. SP, Brazil; 3 Programa de Pós-graduação em Ciência Animal com ênfase em produtos bioativos. Universidade Paranaense, Umuarama, PR, Brazil.

**Keywords:** Leishmania, mutagenic, DNA damage, LDH levels, comet assay, Leishmania, mutagênico, dano ao DNA, níveis de LDH, ensaio cometa

## Abstract

*Leishmania infantum* is a parasite that causes leishmaniasis in its visceral clinical manifestations, which is considered a zoonosis and can infect both humans and animals. Currently, there is no highly effective treatment available, and many animals that exhibit symptoms ultimately die as a result of the disease and its complications. The clinical signs of leishmaniasis are varied and nonspecific. The main symptoms are severe anemia and thrombocytopenia, weight loss, splenomegaly, lymphadenomegaly, liver disease, kidney failure, and skin lesions, among others. Due to the chronic inflammatory state caused by the parasite, an oxidative environment is created, leading to potential cell injury and damage to the infected animals' genetic material. To investigate DNA damage, we conducted the micronucleus test and comet assay, as well as measured serum LDH levels in infected and non-infected dogs. Our results indicate that infected dogs present significantly higher levels of serum LDH (461.4 ± 204.5 U/L, n=36) compared to healthy dogs (142.38 ± 37.94 U/L, n=5). Additionally, the DNA of infected dogs is more damaged than that of the control group, as demonstrated by the micronucleus test (p=0.01) and comet assay (p=0.002). These findings suggest that *Leishmania infantum* infection can lead to clastogenic events, highlighting the need for further research on this process. It is important to consider the potential mutagenic properties of *Leishmania infantum*, given its ability to cause DNA damage in infected animals.

## Resumo

*Leishmania infantum* é um parasita que causa leishmaniose em sua forma clínica visceral, considerada uma zoonose que pode infectar tanto humanos quanto animais. Atualmente, não há um tratamento altamente eficaz disponível, e muitos animais que apresentam sintomas acabam morrendo em decorrência da doença e de suas complicações. Os sinais clínicos da leishmaniose são variados e inespecíficos. Os principais sintomas incluem anemia grave e trombocitopenia, perda de peso, esplenomegalia, linfadenomegalia, alterações hepáticas, insuficiência renal e lesões cutâneas, entre outros. Devido ao estado inflamatório crônico causado pelo parasita, cria-se um ambiente oxidativo que pode levar a lesões celulares e danos ao material genético dos animais infectados. Para investigar o dano ao DNA, realizamos o teste do micronúcleo e o ensaio cometa, além da medição dos níveis séricos de LDH em cães infectados e não infectados. Nossos resultados indicam que cães infectados apresentam níveis significativamente mais altos de LDH sérico (461,4 ± 204,5 U/L, n=36) em comparação com cães saudáveis (142,38 ± 37,94 U/L, n=5). Além disso, o DNA dos cães infectados apresenta mais danos do que o do grupo controle, conforme demonstrado pelo teste do micronúcleo (p=0,01) e pelo ensaio cometa (p=0,002). Esses achados sugerem que a infecção por *Leishmania infantum* pode levar a eventos clastogênicos, ressaltando a necessidade de mais pesquisas sobre esse processo. É importante considerar as potenciais propriedades mutagênicas de *Leishmania infantum*, dada sua capacidade de causar danos ao DNA em animais infectados.

## Introduction

According to the Pan American Health Organization and the World Health Organization (WHO), there are more than 12 million people infected with leishmaniasis worldwide, with 360 million people at risk of contracting the disease (Vilas-Boas et al., 2024). Although leishmaniasis is present on all continents, Brazil accounts for 90% of the reported cases in Latin America. It is estimated that approximately 600,000 cases of visceral leishmaniasis (VL) and 59,000 deaths related to VL occur annually worldwide (Solano-Gallego et al., 2009). Visceral leishmaniasis is a zoonotic disease caused by *Leishmania infantum* (*L. infantum*) (currently considered synonymous with *Leishmania chagasi*), an obligate intracellular parasite that is transmitted through sand fly vectors. VL is the most severe form of leishmaniasis in both humans and dogs.

The primary reservoir of the parasite is the domestic dog, which makes companion dogs a public health concern. High infection rates in dogs are closely associated with an increased risk of the disease in humans (Morales-Yuste et al., 2022). Besides causing VL in humans, *L. infantum* is also the causative agent of canine visceral leishmaniasis (CVL) (Ivănescu et al., 2023).

The life cycle of the parasite begins when dogs are infected with *L. infantum* promastigotes through vector bites, typically from infected female *Lutzomyia longipalpis* (Psychodidae). Subsequently, the promastigote forms invade host cells, primarily macrophages, and replicate as intracellular amastigotes (Silveira et al., 2009). These amastigote forms can then spread to the mononuclear cells of the reticuloendothelial system, including the spleen, liver, and bone marrow, leading to a chronic, severe, and often fatal disease (Silva et al., 2021).

However, the disease does not progress in a significant proportion of animals. They are able to control the parasite infection and live for years, or even their entire lives, without exhibiting any clinical signs. These animals are considered infected but asymptomatic (Podaliri Vulpiani et al., 2011). Resistance or susceptibility to the disease is directly correlated with the induction of a Th1 immune response or a Th2 immune response, the first characterized by the production of IFN-γ, IL-2, and TNF-α, and the latter characterized by the production of IL-4, IL-5, IL-10, IL-13, and TGF-β. The type and level of activation of the immune response are considered decisive factors in determining the severity of the disease (Brodskyn & Kamhawi, 2018).

On the other hand, given the prolonged time of infection, *L. infantum* can weaken the immune system eventually. Within the parasitic infection’s microenvironment, eosinophilic peroxidase (EPO) is activated and generates hypobromous acid (HOBr) from H_2_O_2_ and bromide ions. Peroxidases as macrophage peroxidase (MPO) can also generate nitrogen dioxide (NO_2_) from H_2_O_2_ and nitrite as substrates (Kocyigit et al., 2005; Avila et al., 2025). The mechanisms of nitration, oxidation, and chlorination that are activated to fight parasites can cause injury to host cells and induce oxidative stress on the DNA strand, resulting in DNA breaks if an overproduction of reactive oxygen and nitrogen species (ROS and RNS) occurs.

The DNA damage can be correlated with the parasite infection (Silva et al., 2023). Silva e cols (2023) finds that biomonitoring *Leishmania* infection by micronuclei and comet assay is not possible in medullary lymphocytes obtained by puncture of blood marrow cells (Silva et al., 2023).

In this study, we evaluated cell damage and DNA integrity of peripheral lymphocytes using the micronucleus test and comet assay to best understand the relation between *L. infantum* infection and DNA damage and mutation.

## Materials and methods

### Ethics

The procedures used in this study were approved by the Ethics Committee on the Use of Animals (CEUA) of the Latin American Integration University (UNILA, Foz do Iguaçu, Brazil), under process number 001/2019-CEUA-UNILA.

### Animals

For this study, peripheral blood samples were collected from a total of 41 dogs of different breeds, including five healthy non-infected control dogs (NIC) and 36 asymptomatic dogs naturally infected with *Leishmania infantum* (LID), serving as the Leishmania-infected group. All animals were companion dogs residing in Foz do Iguaçu, PR, Brazil. Blood samples were collected by a veterinarian using vacutainer tubes containing heparin or EDTA as anticoagulants. *Leishmania infantum* infection was confirmed through immunochromatographic test using kit SNAP 4DX (IDEXX, Maine, EEUU) and laboratory indirect fluorescent antibody test (IFAT) (Tecsa Laboratory, Minas Gerais, Brazil).

### Micronucleus assay

To assess chromosomal damage in infected dogs, we performed the Micronucleus (MN) Test on binucleate (BN) lymphocytes cells. The methodology used was adapted from the proposed protocol by Backer et al. (2001), Dönmez-Altuntas et al. (2006) and Fenech (2007).

In summary, 150 μL of heparinized blood samples were collected from NIC, LID, and one MN positive control group incubated with cyclophosphamide (Merck KGaA, Darmstadt, Germany) (NIC + CPM). The samples were then cultured for 72 hours at 37 °C in 5 mL of RPMI-1640 culture medium (Vitrocell, Campinas, SP, Brazil). The culture medium was supplemented with 0.5 mL of fetal bovine serum (Vitrocell, Campinas, SP, Brazil), 0.1 mL of phytohemagglutinin (Vitrocell, Campinas, SP, Brazil), 100 U/mL penicillin, and 100 μg/mL streptomycin (Vitrocell, Campinas, SP, Brazil). After 8 hours, 1.25 μL of cyclophosphamide (Sigma-Aldrich, Melville, NY, USA), at a concentration of 50 μg/mL, was added to the NIC + CPM group. At 44 hours of incubation, cytochalasin-B (Sigma-Aldrich, Melville, NY, USA) was added to the cultures at final concentration of 3 μg/mL. The cultures were stopped at 72 hours, treated with a hypotonic solution (0.075 M KCl) for 3 minutes, and fixed in two changes of methanol-acetic acid (3:1 v/v). The fixed cells were spread onto glass slides and stained with 5% Giemsa (Laborclin, Pinhais, PR, Brazil) for 7 minutes. All slides were coded and read blindly. Cells with two nuclei surrounded by cytoplasm and a cell membrane were observed and scored for the presence of micronuclei (MN) using a NO216B microscope (Global Optics, Monte Alto, SP, Brazil) at a total magnification of 1,000X. The number of MN in 1,000 binucleate (BN) cells was counted, and the frequency of MN per 1,000 BN cells was calculated for each dog. Published criteria for scoring MN in BN cells and selecting BN cells were followed (Fenech, 1993, 2007). Statistical analyses were through the Kruskal-Wallis test followed by Dunn's post-hoc test, both with significance levels of 5%. These analyzes were performed using the GraphPad Prism version 8 software (GraphPad Software, Inc.).

### Comet assay

Clastogenic effects in nucleated cells from whole blood of *L. infantum*-infected dogs were assessed using the alkaline comet assay, renowned for its high sensitivity in detecting both single-strand DNA breaks (SSBs) and double-strand DNA breaks (DSBs) (Araldi et al., 2015).

Slide preparation: Slides measuring 26 × 76 mm were dipped in a solution of normal melting point agarose (NMA) (Sigma-Aldrich, Melville, NY, USA) diluted in PBS to a concentration of 1.5% at 60 °C. One side of the slides was cleaned with a paper towel. The slides were then dried horizontally overnight.

Sample preparation: A volume of 200 μL of whole blood in EDTA from NIC and LID was added to 200 μL of RPMI-1640 medium and incubated for 1 hour at 37 °C. Then, the samples were centrifuged at 10,200 x g for one minute, and the supernatant was discarded. A volume of 10 μL of the pellet was transferred to 0.2 mL tubes and mixed with 75 μL of low melting point agarose (LMA) at 37 °C. The final volume of the cell suspension was adjusted to 85 μL, and it was transferred to pre-coated NMA slides. The slides were covered with coverslips and maintained at 4 °C for 20 minutes. The coverslips were gently removed, and the slides were placed in a Coplin jar containing lysis solution (2.5 mM NaCl, 100 mM EDTA, 10 mM Tris-HCl, 1.1% Triton X-100, and 11.2% DMSO) at 4 °C, remaining in it for one hour. From this step, the work was conducted with the laboratory lamps turned off to prevent the induction of DNA damage.

Electrophoresis: After lysis, the slides were washed with PBS and transferred to an electrophoresis tank, where they were covered with an electrophoresis buffer with a pH greater than 13.0 (300 mM NaOH and 1 mM EDTA) at 4 °C for 40 minutes. After this time, the electrophoretic run was performed with a current of 25V (0.86 V/cm) and 300 mA for 20 minutes to facilitate the migration of free DNA fragments. The slides were then transferred to a Coplin jar containing a neutralizing buffer (400 mM Tris-HCl, pH 7.5) for five minutes. The material was fixed in absolute ethanol for five minutes.

The slides were stained with a 20 μL of a 20 μg/mL propidium iodide (PI) solution and analyzed under an epifluorescence microscope (Nikon 80i, Nikon Instruments Inc, Melville, NY, USA) equipped with an excitation filter of 510-560 nm and a barrier filter of 590 nm, at a total magnification of 400X. A total of 100 nucleoids were analyzed per slide, and they were assigned scores ranging from 0 (no DNA damage) to 2 (maximum DNA damage). The scores were obtained by summing the product of the observed number of nucleoids per category by their respective category value. Statistical analysis was performed using the Mann-Whitney test with a significance level of 5%, using GraphPad Prism version 8 software (GraphPad Software, Inc.).

### Determination of serum lactate dehydrogenase (LDH)

To evaluate systemic cell damage, serum LDH activity was assessed. Serum samples were incubated with the Lab Test LDH liquiform commercial kit (Labtest, Vista Alegre, MG, Brazil) (Passonneau & Lowry, 2008). The absorbance values were measured using a semi-automatic biochemical analyzer, Bio 200S (Bioplus, São Paulo, Brazil), at a wavelength of 340 nm. The results were expressed as U/L and a statistical comparison was performed by one-way ANOVA followed by Bonferroni's multiple comparisons Post Hoc test.

## Results

### Micronuclei assay

**Figure 1:**
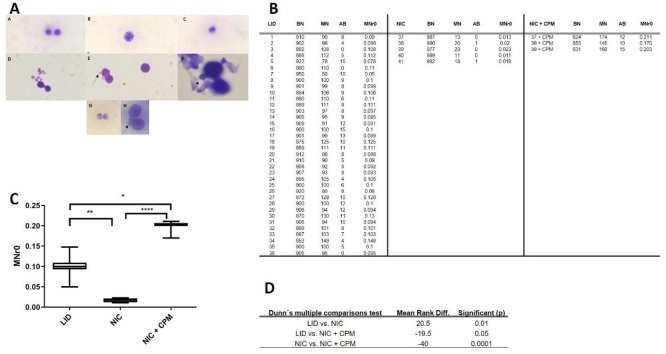
Results of micronucleus assay. (A) Representative figures of the MN assay slide; A.A and C: Binucleated cells; A.B Mononucleated cell; A, D, E and G anaphasic bridges (arrows); A.F and H binucleated cells contain micronucleus (arrows); (B) Table with the absolute values of MN (mononucleated cells) and BN (binucleated cells) per animal, and the micronucleus frequency (MNr0); (C) Graphical comparison of groups; (D) Statistical p values of Dunn’s multiple comparison test. LID = Leishmania-infected dogs; NIC = non-infected control; NIC + CPM = non-infected control + cyclophosphamide. [[Q3: Q3]]

A total of 1,000 BN lymphocytes cells were analyzed per animal. Representative photographs are presented in Figure 1A. Binucleated cells, which are essential for evaluating micronuclei formation, were frequently identified (Figure 1A.A and 1A.C). Mononucleated cells were also present (Figure 1A.B), serving as internal controls for cellular morphology. Structures compatible with anaphase bridges, indicative of chromosomal instability and missegregation events, were identified in several images (Figure 1A.A, 1A.D, 1A.E, and 1A.G), as indicated by the arrows. Additionally, binucleated cells containing micronuclei—key markers of genotoxic damage—were clearly visualized (Figure 1A.F and 1A.H), also marked with arrows. The calculated frequency of micronuclei (MN r_0_) is shown in the table in Figure 1B. The relative MN frequencies in LID dogs (0.101 ± 0.016) were compared with those in NIC dogs (0.017 ± 0.005) using the Dunn test. A significant difference in MN frequency was observed between LID and NIC dogs (p < 0.01). The positive control group treated with cyclophosphamide (0.195 ± 0.021) also showed significant differences when compared to both the LID group (p < 0.05) and the NIC group (p < 0.0001). The results of all statistical comparisons are presented in Figure 1C and 1D.

### Comet assay

**Figure 2:**
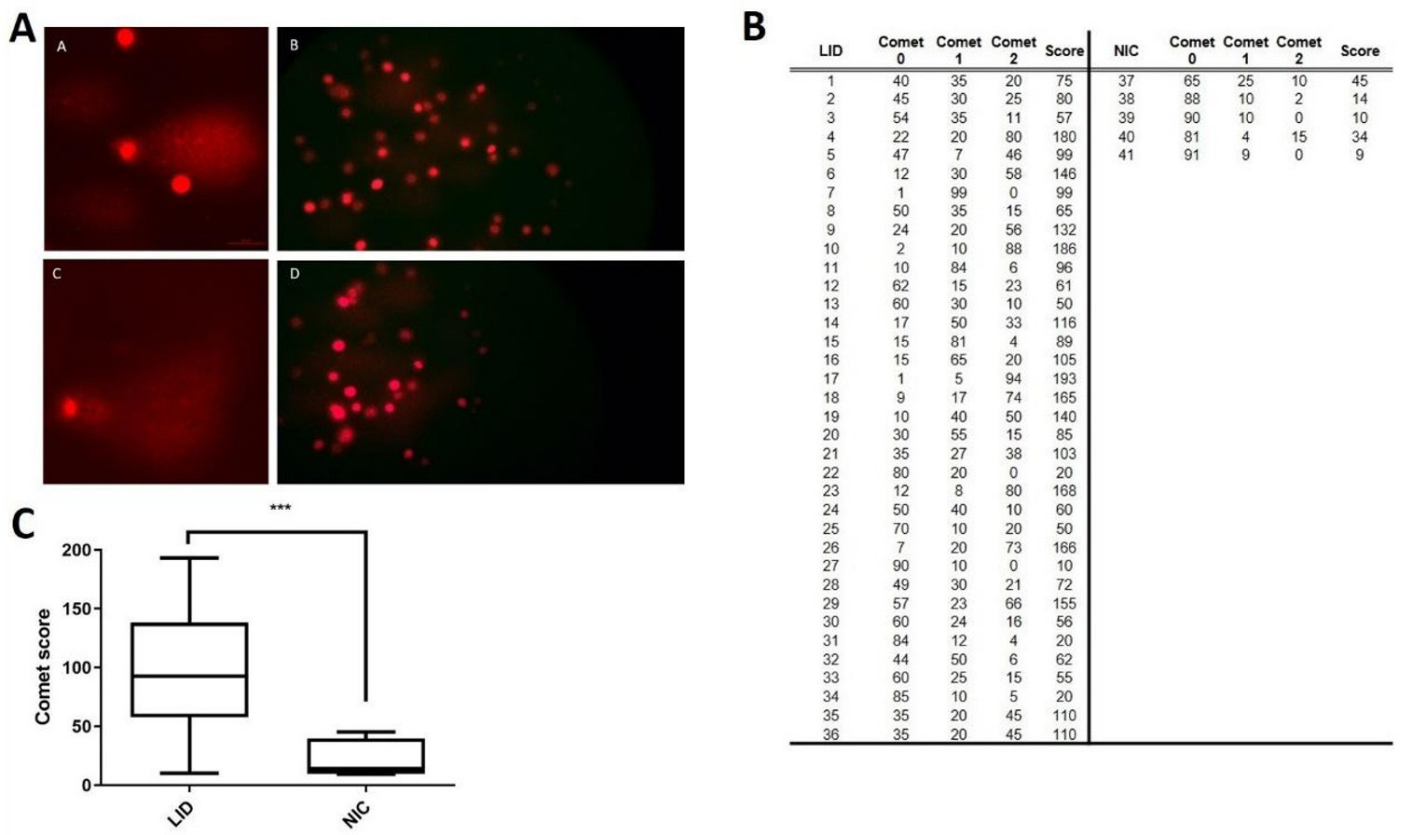
Results of comet assay. (A) Representatives figures of comets; A.A and A.C: comet with damage 1; A. B and A.D: comet with damage 0; (B) Table with the absolute values of comets classified by degree (0: without damage, 1: intermediary DNA damage and 3: maximal DNA damage) and the calculated scores; (C) Graphical comparison of groups by Mann-Whitney test with p=0.002. LID = Leishmania-infected dogs; NIC = non-infected control.

The comet scores were determined qualitatively based on the nucleus-to-tail ratio, and representative images are shown in Figure 2A. The comet scores were calculated as described in the Comet Assay methods section, and the values are presented in the table (Figure 2B). The Mann-Whitney test revealed a statistically significant difference between NIC (score: 90 ± 50.5) and LID (score: 22.4 ± 16.2) groups (p = 0.0002), indicating an increase in clastogenic events in infected dogs compared to baseline values observed in non-infected dogs. Representative comet assay images (Figure 2B), the classification criteria for comet scoring (Figure 2C), and a dot plot comparing NIC and LID groups (Figure 2D) are included in Figure 2.

### LDH quantification

**Figure 3:**
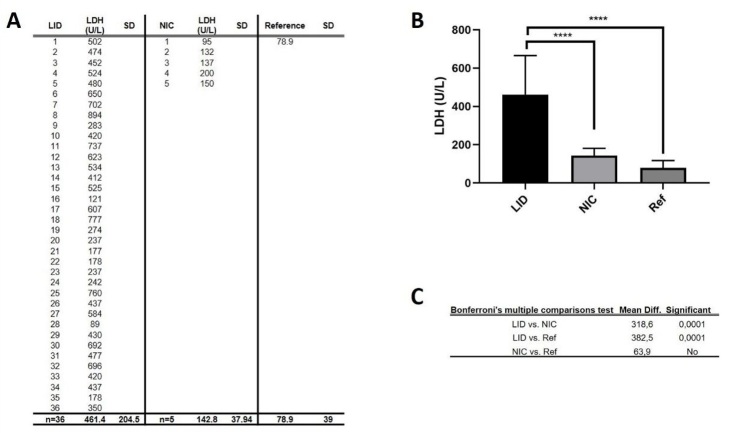
Results of comet assay. (A) Table with the absolute values of LDH concentration in serum; (B) Graphical comparison of groups; (C) The statistical p values of Bonferroni’s multiple comparison test. LDH = lactate dehydrogenase; LID = Leishmania-infected dogs; NIC = non-infected control.

The plasma levels of LDH quantified for NIC and LID are represented in Figure 3A, along with the LDH reference value (Kopanke et al., 2018).

The results in Figure 3B indicate a significant statistical difference between the LID and NIC groups (p = 0.0001) and between the LID group (p = 0.0001) and the reference values, as determined by Bonferroni's multiple comparisons test. The NIC group falls within the range of the reference values, indicating the healthy state of the animals in the control group.

## Discussion

The World Health Organization (WHO) classified leishmaniasis as a zoonosis that has been spreading to areas previously considered safe. It is a zoonosis of great epidemiological importance in human and veterinary medicine caused by a parasite *Leishmania* sp. and its serotypes, with dogs being an important reservoir of the disease. The city of Foz do Iguaçu (Brazil), is an endemic region for *Leishmania infantum* and presents a large number of new cases in dogs daily. According to the guideline published by Solano-Gallego et al. (2009), it is clear that leishmaniasis is a chronic disease capable of causing varied symptoms in animals as well as capable of being asymptomatic for a long time. After escaping the macrophages and immune response, the parasite targets the lymphoid organs, entering the bloodstream. This leads to the variability of clinical symptoms or the presence of subclinical disease.

In view of this, we infer that it is extremely important to establish the relationship between chronic infectious diseases and their possible relationship with other diseases and thus establish early diagnosis and suggest reasons for improvements to the authorities, communities, and animal guardians, in relation to the prevention of the infection.

Leishmaniasis is a disease that can evolve to a chronic status, capable of causing a variability of symptoms and signs that can be observed through clinical and laboratory tests, changes such as leukocytosis or leukopenia (by bone marrow exhaustion) are observed, as well as elevated levels of LDH, C-reactive protein, fibrinogen and normochromic anemia characteristic of chronic inflammation (Solano-Gallego et al., 2016). Statistically significant alterations in serum LDH levels were observed in Leishmania-positive dogs (Figure 3B), suggesting substantial cellular damage and enzymatic leakage into the plasma as a result of cell death, consistent with the findings of Gallo et al. (2015) and Yu et al. (2017). This result is particularly relevant as it indicates that systemic biochemical alterations and potential comorbidities in infected dogs are not merely coincidental, but may instead be directly associated with the chronic pathological processes induced by leishmaniasis.

Then, the persistent infection can develop an inflammatory state that becomes a chronic process with associated cell damage. Although biological carcinogenic agents are primarily restricted to viruses, IARC (The International Agency to research in Cancer) classified *Schistosoma haematobium*, *Opisthorchis viverrini*, *Clonorchis sinensis* and *S. japonicum* as carcinogenic parasitic infections. Furthermore, the relationship between inflammation and cancer has been confirmed by numerous epidemiological observations (Schwing et al., 2019). Such chronic inflammation can promote carcinogenesis through oxidative stress mediated by TNF-alpha (tumor necrosis factor), interleukins, interferons and macrophages, together stimulating reactive oxygen and nitrogen species (ROS and RNS) production capable of generating breaks in the DNA strand (Kocyigit et al., 2005; Meira et al., 2008; Schwing et al., 2019).

When DNA is under attack, mutations can be accumulated and inflammation can become a factor in initiating and in promoting carcinogenesis. We found through micronucleus and comet assays that normal dogs have basal DNA damage, however we observe that, in dogs with leishmaniasis, the damage to DNA is statistically significant (Figure 1C and Figure 2C).

We cannot affirm that *L. infantum* is a biological carcinogen, but acknowledging that chronic infections caused by biological agents contribute to approximately 20% of cancer causes in the world (this fraction may vary according to regions) (Basu, 2018), this case of chronic DNA damage may be the first sign of the carcinogenic process. Studies reported leishmaniasis as a probable cause of carcinomas (Chisti et al., 2016) and lymphomas (Ferro et al., 2013; Masrour-Roudsari & Ebrahimpour, 2017), but it is worth mentioning that the tissue under the condition of chronic inflammation, since *Leishmania* sp. is capable of causing important inflammation in the skin, is characterized by a high rate of cell proliferation, so the tissue is repaired but this persistent proliferation increases the rate of random mutations triggered by oxidative stress, in addition to perpetuating damage to the cell genome.

Given the evidence of DNA damage in dogs infected with Leishmania infantum, studies assessing in vivo DNA damage, particularly those evaluating anti-Leishmania drugs (Moreira et al., 2017), should take this finding into account. The presence of substantial DNA damage in infected hosts challenges the use of MN and comet assays for drug evaluation under active infection conditions. One of the limitations of this study include the discrepancy in sample size between the control group (n = 5) and the group of dogs infected with *L. infantum* (n = 36). Although this imbalance could potentially affect the statistical power, we mitigated this issue by applying non-parametric statistical tests appropriate for unequal group sizes. Additionally, PCR was not performed in the control group to definitively exclude subclinical infection. While we used two combined serological tests to increase diagnostic reliability — an approach supported by surveillance protocols and prior literature — this remains a relevant limitation. Future studies with more balanced sample sizes and the inclusion of molecular diagnostics are encouraged to confirm and expand upon these findings.

In summary, we found DNA damage in peripheral blood cells of dogs infected with *Leishmania infantum* as well as elevated levels of LDH. We encourage new studies on the role of *Leishmania* sp. in chronic infections and carcinogenesis.
